# Validation of two predictive models for survival in anaplastic thyroid cancer (ATC)

**DOI:** 10.1186/s12885-024-13217-2

**Published:** 2024-11-29

**Authors:** Lukas Käsmann, Alexander Nieto, Robert Rennollet, Ralph Gurtner, Dmytro Oliinyk, Teresa Augustin, Viktoria Florentine Koehler, Maria Neu, Claus Belka, Christine Spitzweg, Josefine Rauch

**Affiliations:** 1 Department of Radiation Oncology, University Hospital, LMU Munich, Munich, 81377 Germany; 2 German Cancer Consortium (DKTK), Partner Site Munich, Munich, 80336 Germany; 3 Department of Hematology and Oncology, Klinikum rechts der Isar der TU München, Ismaninger Straße 22, Munich, 81675 Germany; 4 Department of Plastic, Hand and Reconstructive Surgery, University Hospital Regensburg, Franz-Josef-Strauss-Allee 11, Regensburg, 93053 Germany; 5 Department of Medicine IV, LMU University Hospital, LMU Munich, Munich, 81377 Germany; 6 Department of Radio-Oncology, University Hospital of Augsburg, Augsburg, Germany; 7 Bavarian Cancer Research Center (BZKF), Munich, Germany; 8 Adjunct Academic Appointment, Division of Endocrinology, Diabetes, Metabolism and Nutrition, Mayo Clinic Rochester, Rochester, MN USA

**Keywords:** Anaplastic thyroid cancer, Survival, Score, Prediction, Multimodal treatment

## Abstract

**Background:**

The prognosis of patients with anaplastic thyroid cancer (ATC) remains dismal. A small portion of patients experience longterm survival and need to be identified before treatment allocation. Survival scores may guide clinicians making more informed decisions about treatment options and improve the understanding of patients’ prognosis. The aim of this study was to validate two prognostic scores using an independent dataset to analyze which prognostic index is superior in discriminating survival.

**Methods:**

Thirty-four patients with histologically confirmed ATC diagnosed between January 2009 and December 2019 were consecutively treated at our department and evaluated. Next generation sequencing was performed in 7 (21%) patients, but no druggable mutation was found. 50% of all patients received surgery and 56% were treated with chemoradiotherapy. The median radiation dose in equivalent dose in 2 Gy fractions (EQD2) was 50 Gy (SD:21 Gy). The study compared the discrimination of the Sugitani Prognostic Index (SPI) and the Marchand-Crety Prognostic Score (MCPS) using concordance statistics, area under the receiver-operating characteristics curve (AUC), net reclassification index, and integrated discrimination improvement for 6-month survival.

**Results:**

The median survival of the entire cohort was 5 months (range: 1-133). The AUC for 6-month survival was 0.85 (95% confidence interval [CI]:0.72–0.97) for SPI and 0.69 (95% CI: 0.56–0.83) for MCPS (*p* < 0.0001). Using the net reclassification index (NRI), 73% of patients were correctly reclassified using SPI instead of MCPS for 6-month survival (*p* = 0.0237).

**Conclusion:**

The SPI was more accurate than the MCPS to determine patients’ life expectancies and should be recommended for clinical guidance and treatment allocation. In the last decade, comprehensive genetic profiling of actionable mutations in ATC has become vital to guide targeted therapy.

## Background

Despite significant progress in the therapeutic management of patients with anaplastic thyroid cancer (ATC), it is still associated with a dismal prognosis and treatment options are limited. Median survival time of conventional treatment is 5 months (range: 2–6) [1–5]. However, the introduction of immune checkpoint inhibition, targeted therapy and intensified therapy combination has significantly improved outcomes [6–8]. To identify the appropriate treatment regime, survival scores can guide clinicians in treatment-decision making, especially in patients with a short remaining life span. Therefore, it is essential to accurately predict patients’ prognosis based on reliable survival scores. Not all patients may tolerate aggressive multimodal treatments and therefore clinical scores may identify patients in need of early palliative or best supportive care. Currently, only two survival scores have been developed but their prognostic value is unknown due to the lack of external validation. In consequence, we investigated both tools to determine patients’ life expectancies and identify which score is more reliable to predict the outcome of ATC patients.

## Methods

In this retrospective study all consecutive patients with histologically or cytologically confirmed ATC diagnosed between January 2009 and December 2019 and treated at our department were enrolled. Patient and treatment characteristics are displayed in Table 1.

**Table 1:** Patient and treatment characteristics

	**N**	**%**
**Age at diagnosis**		
≤75 years	23	68
>75 years	11	23
**Gender**		
Female	19	56
Male	15	44
**T category (TNM)**		
1-3	5	15
4	29	85
**Tumor size**		
<5cm	15	44
≥5cm	19	56
<7.5cm	24	71
≥7.5cm	10	29
**Nodal involvement**		
Yes	23	68
No	11	32
**Metastasis at diagnosis**		
Yes	20	59
No	14	41
**UICC stage**		
**IVA**	2	6
**IVB**	12	35
**IVC**	20	59
**Karnofsky Performance Status**		
≥80%	21	62
<70%	13	38
**Acute symptoms**		
Yes	22	65
No	12	35
**Leucocytosis**		
Yes	12	35
No	22	65
**Surgery**		
Yes	17	50
No	17	50
**Concurrent chemotherapy**		
Yes	19	56
No	15	44
**Cumulative dose of definitive radiotherapy in EQD2**		
≤50Gy	17	50
>50Gy	17	50

*EQD2 *equivalent dose in 2 Gy fractionsm, *TNM *TNM Classification of Malignant Tumors

Median age at diagnosis was 71 years (range: 51–97) and gender ratio 1:3 in favor for females. Median tumor size was 6 cm (range: 1.2–11.5). Acute symptoms defined as duration of complaints less than 1 month were present in 65% of all patients. The institutional review board at Ludwig-Maximilians-University (Munich, Germany) reviewed and approved the retrospective study protocol (approval number: 20–023).

The diagnosis of ATC was histologically confirmed in all patients and diagnosed as stage IV according to the revised 8th edition of the Union for International Cancer Control (UICC) TNM [9]. For patients diagnosed before publication of the 8th edition of the UICC TNM, patients were regrouped accordingly. Surgery was performed in seventeen (50%) patients depending on UICC stage, extent of tumor invasion and performance status. A hemithyroidectomy was performed in six (18%) patients due to limited tumor invasion and nine (26%) patients had received a total thyroidectomy. A subtotal thyroidectomy was performed in two (6%) patients. A microscopically margin-negative resection was achieved in one (3%) patient (R0) and the removal of all macroscopic disease was performed in nine (26%) patients. Macroscopic residual tumor after surgery was found in seven (21%) patients (R2).

Routine next generation sequencing (NGS) has been implemented in the diagnostic workup towards the second half of the observation period, therefore only 7 (21%) patients received further molecular diagnostics, but no druggable mutation was found.Three-dimensional conformal radiotherapy (3D-CRT) technique was administered in the majority of patients (*n* = 21, 62%). However, 2 (6%) and 11 (32%) patients received intensity modulated radiation therapy (IMRT) and volumetric modulated arc therapy (VMAT), respectively. The median radiation dose in equivalent dose in 2 Gy fractions (EQD2) was 50 Gy (SD: 21 Gy).

Concurrent chemoradiotherapy was administered to 19 patients (56%). Of these, 9 patients (47%) received carboplatin/paclitaxel chemotherapy, 7 patients (37%) received doxorubicin, and 2 patients (11%) received cisplatin/doxorubicin. Additionally, 4 patients (12%) received sequential chemotherapy with carboplatin/paclitaxelcombination or doxorubicin. Nine (27%) patients received radiotherapy alone due to performance status deterioration and were provided with best supportive care following local radiotherapy.

Prognostic groups according to SPI and MCPS (see Table 2) were calculated.

**Table 2:** Prognostic factors according to Sugitani et al. and Marchand-Crety et al. and the corresponding scoring points [8, 9]

*Sugitani Prognostic Index (SPI)* [8]		*Marchand-Crety Prognostic Score (MCPS)* [9]	
*Prognostic factors*	*Scoring points*	*Prognostic factors*	*Scoring points*
**Metastases at diagnosis**		**Metastases at diagnosis**	
Yes	1	Yes	1
No	0	No	0
**Acute symptoms (<1 months)**		**Age**	
Yes	1	≤75 years	0
No	0	>75 years	1
**Tumor size**		**Tumor size**	
≤5cm	0	≤7.5cm	0
>5cm	1	>7.5cm	1
**Leukocytosis (white blood cell count >10,000/mm**^**3**^**)**			
Yes	1		
No	0		

Statistical analyses were performed using SPSS statistics 28 (IBM, New York City, NY, SA) and R v4.2.1. The “glm” function of the package “stats” v4.2.1 was used to calculate simple logistic regression models and thereof derived metrics. The “concordance index” function of the package “survcomp” v1.46.0 was used to calculate the concordance index. The “roc-test” function of the package “pROC” v1.18.0 was used to calculate De Long´s test for AUC of the receiver operating characteristic curve (ROC) comparison. Evaluation metrics of the AUC of the ROC were computed by the “cutpointr” function of the package “cutpointr” v1.1.2 with default values and 1000 bootstrap runs whenever bootstrap confidence intervals are supplied. The plot of superimposed AUC of the ROC of the models used was generated by the “plotROC” function of the package “predictABEL” v1.2–4. The Continuous Net Reclassification Improvement (NRI) with 95% CI and p-value of the test and Integrated Discrimination Improvement (IDI) with 95% CI and *p*-value of the test were calculated using default values with the “reclassification” function of the package “predictABEL” v1.2–4.

## Results

Patient and tumor characteristics are shown in Table 1. Regarding age at diagnosis, 23 (68%) patients were ≤ 75 years old, while 11 (32%) patients were above 75 years old. In terms of gender, 19 (56%) patients were female, and 15 (44%) patients were male. The T category analysis of the TNM classification showed that 5 (15%) patients belong to the categories 1–3, while 29 (85%) patients were classified as T category 4. Tumor size was divided into two groups: 15 (44%) patients had tumor sizes < 5 cm, and 19 (56%) patients had tumor sizes ≥ 5 cm. In relation to nodal involvement, 23 (68%) patients had positive nodal status, while 11 (32%) patients had negative nodal involvement. Metastases at diagnosis were found in 20 (59%) patients, while 14 (41%) patients did not have metastases. Evaluating the Karnofsky Performance Status, 21 (62%) patients had a status of 80% or higher, while 13 (38%) patients had a status below 70%. Acute symptoms were reported in 22 (65%) patients, while 12 (35%) patients did not experience symptoms. Concurrent chemotherapy was administered to 19 (56%) patients, whereas 15 (44%) patients did not receive it simultaneously.

The median overall survival (OS), 6- and 12- month survival rate of the entire cohort was 5 months (range: 1–133), 41% and 19%, respectively.

In univariate analysis, tumor size (< 5 cm, χ2 = 8.448, *p* = 0.004; <7.5 cm, χ2 = 14.648, *p* < 0.001), absence of metastatic disease (χ2 = 16.311, *p* < 0.001), Karnofsky Performance Status (≥ 80%, χ2 = 19.137, *p* < 0.001), no leucocytosis (χ2 = 13.281, *p* < 0.001), no acute symptoms (χ2 = 13.503, *p* < 0.001), surgery (χ2 = 23.775, *p* < 0.001) and a cumulative radiation dose of definitive radiotherapy in equivalent 2 Gy fractions (EQD2 > 50 Gy, χ2 = 13.064, *p* < 0.001) were associated with improved OS (see Table 3).

**Table 3:** Uni- and multivariate analysis for OS

	3-month survival rate	6-month survival rate	12-month survival rate	Univariate	Multivariate
**Age at diagnosis**					
≤75 years	71	44	26	0.155	
>75 years	55	36	0		
**Gender**					
Female	68	53	18	0.421	
Male	47	27	20		
**T category (TNM)**					
1-3	100	60	40	0.178	
4	52	38	15		
**Tumor size**					
<5cm	87	60	40	**0.004**	0.819
≥5cm	37	26	0		
**Tumor size**					
<7.5cm	75	54	27	**<0.001**	0.858
≥7.5cm	20	10	0		
**Nodal involvement**					
Yes	52	35	10	0.067	
No	73	55	36		
**Metastasis at diagnosis**					
Yes	30	15	5	**<0.001**	**0.035**
No	100	79	39		
**Karnofsky Performance Status**					
≥80%	81	62	31	**<0.001**	0.959
<70%	23	8	0		
**Leucocytosis**					
Yes	25	8	0	**<0.001**	0.302
No	77	59	30		
**Acute symptoms**					
Yes	41	23	0	**<0.001**	0.066
No	92	75	50		
**Surgery**					
Yes	94	71	35	**<0.001**	**0.034**
No	24	12	0		
**Concurrent chemotherapy**					
Yes	68	42	16	**0.847**	
No	47	40	24		
**Cumulative dose of definitive radiotherapy in EQD2**					
≤50Gy	29	24	0	**<0.001**	0.464
>50Gy	88	59	39		

*EQD2 *equivalent dose in 2 Gy fractions, *TNM *TNM Classification of Malignant Tumors

These factors were evaluated in logistic multivariate analysis and two independent risk factors remained: namely meta-static disease (hazard ratio [HR] = 0.3.811, 95%CI = 1.097–13.242, *p* = 0.035) and surgery (HR = 0.255, 95%CI = 0.072–0.902, *p* = 0.034).

We sought to identify the model that best predicts the binary response of OS at 6 months.

We first used a binomial logistic regression model to analyze the relationship between the binary dependent variable OS at 6 months and the risk group calculated according to SPI or MCPS survival score. The logistic regression model using SPI risk groups performed better than the MCPS with an odds ratio of 6.23 (95% CI 2.3–23.1; *p* = 0.0014) compared to 4.78 (95% CI 1.36–34.17; *p* = 0.0514). The area under the ROC curve was significantly higher in the model using SPI risk groups compared to the MCPS (AUC of the ROC 0.85 vs. 0.69; DeLong´s test *p* = 0.02265). The optimal groups for binary 6-month OS prediction were calculated for each regression model. Patients with the SPI risk groups 0 and 1 had an increased probability for survival at 6 months. Patients with a MCPS survival score of 0 and 1 had an increased probability for survival at 6 months.

The SPI risk group model exhibited a higher concordance index (C-index) of 0.88 (95% CI: 0.81–0.95; 1.36 × 10–24). This indicates a strong discriminatory power of the model in ranking individuals according to their 6-month OS rate. The MCPS model demonstrated a slightly lower concordance index of 0.81 (95% CI: 0.71–0.92; *p* = 3.45 × 10 − 9) compared to the SPI risk groups model. Although lower, this concordance index still indicates a reasonably good predictive ability of the model.

The NRI is a measure that quantifies the improvement in risk prediction achieved by one model compared to another model. The NRI evaluates the ability of a new model to correctly reclassify individuals into appropriate risk categories compared to a reference model with positive values indicating an overall improvement and negative values a reduction in risk prediction. It provides an assessment of the extent to which the new model improves risk stratification. We calculated the NRI on abovementioned logistic regression models. The NRI favored the SPI risk group model over the MCPS model in 6-month OS prediction (NRI = 0.73; 95% CI 0.10–1.36; *p* = 0.0237). The IDI comparing the logistic regression models suggests that the difference in average predicted risks for survival at 6 months was increased by 23% using SPI Risk Groups compared to the MCPS (95% CT 9.0–38%; *p* = 0.0018).

These metrics assess the overall performance of the logistic regression models. Patients with a high MCPS had 0% probability of survival at 6 months in our data set while patients with high risk according to Sugitani et al. had a 7% probability of 6-month survival (Table 4).

**Table 4:**
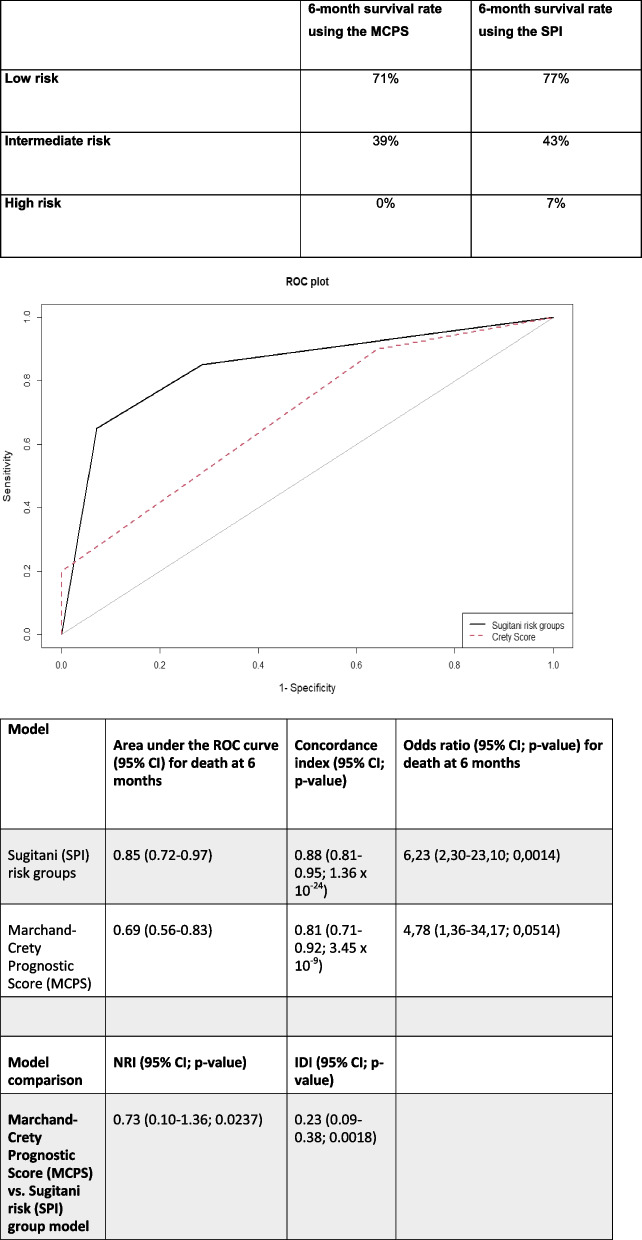
Risk groups according to survival score and 6-month survival rate

## Discussion

In this study, we conducted a comparison between two survival scores, namely SPI and MCPS, to assess their accuracy in determining life expectancies of patients with non-oncogene-addicted ATC in an independent patient cohort. Our findings indicate that the SPI demonstrates greater accuracy compared to the MCPS in determining patient’s prognosis. Therefore, we recommend the use of SPI for clinical guidance and treatment allocation.

In the last decade, several new treatment combinations and treatment intensifications as well as routinely molecular genetic testing such as NGS on tumor samples have been introduced in the management of ATC. However, the outcome of ATC patients without driver mutations remains poor with a 6-month survival rate ranging between 30 and 40% similar to our findings [1, 2, 4]. Longterm survival (≥ 2 years) with ATC is rare and may be achieved in BRAF V600E-mutant ATC treated with dabrafenib/trametinib or in non-oncogene addicted ATC after complete resection followed by adjuvant chemoradiation [5, 10]. However, not all patients are suitable for such intensive treatment approaches [11]. In this situation, clinical guidance and treatment allocation are crucial. Therefore, it is vital to precisely determine a patient’s prognosis through reliable and validated survival scores. This ensures proper treatment allocation, whether the patient benefits from an intensive multimodal approach or requires palliative/best supportive care. Nethertheless, NGS testing should always be recommended and may reveal druggable targets such as BRAFV600E-mutation offering effective and well tolerated treatment options [5, 12]. In the US, BRAF mutations are reported in 40–45% of cases [4, 13], while European studies report a lower range of 14–37% [14, 15]. South Korean data shows a 41% BRAF alteration rate in a cohort of 13 ATC patients [16]. In our cohort, NGS testing was not routinely performed due to the observation period starting in 2009. However, no druggable mutation was found in this subgroup (*n* = 7, 21%).

Several potential prognostic factors in the treatment of ATC have been suggested such as performance status, metastatic involvement, age, early onset of symptoms and operability [3, 17–20]. The prognostic value of age is quite controversial due to different cutoff levels and the potential impact of comorbidities or the performance status which cause an additional bias. An analysis of the Surveillance, Epidemiology, and End Results (SEER) database with 586 patients found that patients aged ≤ 70 years have a better prognosis compared to older patients [21]. Their finding mainly states that younger patients are eligible to more intensive treatment modalities due to higher organ reserves. According to Crety et al., age is a strong independent prognostic factor, and patients above the age of 75 have a significantly lower survival time [18]. Surprisingly, Sugitani score found no association of age with overall survival and therefore did not include age in their prognostic index [17].

In addition, tumor size and distant metastases are usually relevant prognostic factors for OS. Several studies show that patients with small intrathyroidal tumor masses and no further extension to the capsule or distant metastasis may profit from multimodal therapies including radical surgery, chemoradiation and adjuvant systemic treatment [20, 22]. For patients with stage IVB ATC, aggressive surgery leads to better outcomes than limited surgery [23]. Besides, several studies show that surgery followed by radiotherapy achieve better results compared to surgery alone [24, 25]. Furthermore, the addition of chemotherapy to radiotherapy results in an improvement of OS independently from surgical resection and metastatic disease based on a SEER analysis [26]. Nevertheless aggressive therapies must be chosen carefully, as the patient’s quality of life, side effects, and chances of therapy must be weighed case by case.

The majority of ATC patients show an early onset of acute symptoms like hoarseness, dysphagia, or dyspnea, which may be associated with higher rates of treatment complications/side effects and worse median survival time [18, 23, 27]. These symptoms might be caused by rapid tumor progression or infiltration of surrounding organs and tissue. Importantly, more radical surgical methods in patients with advanced tumor disease including total laryngectomy, esophagectomy, and/or resection of the great vessels have not shown significant survival benefit [28]. As a result, only selected patients should be considered for this aggressive treatment approach. Best supportive care should always be considered in patients with advanced cancer. In addition, early tracheostomy can be used to relieve symptoms but also has negative effects on the patient’s quality of life (QoL) and must therefore be critically discussed with the patient.

The predictive and prognostic role of inflammation in ATC is unknown. Sugitani et al. proposed that the white blood cell count (WBC) is a prognostic biomarker and found significantly worse survival in patients with high WBC in contrast to Marchand-Crety et al. [17, 18]. In addition, recent studies found that the neutrophil-to-lymphocyte ratio (NLR) could serve as a prognostic factor and response marker in ATC, where an NLR increase is associated with a worse prognosis [29]. The Marchand Crety-score did not include NLR due to missing data (56%) [18]. However, it is essential to highlight that WBC and NLR count alone are not precise indicators of ATC outcomes and should be weighed alongside other clinical and pathological parameters.

Both ATC survival scores have value for clinicians due to their ease of use. When creating the Crety Score, a larger patient cohort was included than in Sugitani et al. [17, 18]. However, a major problem is that up to 56% of the data was lost for some patients. Furthermore, a small subset of ATC patients is thought to have a prior or coexistent differentiated thyroid carcinoma [5]. Unfortunately, this was not taken into account when creating the Crety score [18]. In our study the Sugitani score proved to be more accurate in predicting the patients survival length. Furthermore, the Sugitani Score has already been further investigated by the same working group and institution [30].

There are several limitations to our study that should be mentioned. The study design is retrospective with the possibility of selection bias and the small sample size of 34 participants which may limit the generalizability of our results. NGS testing was conducted only in a minority of the patient cohort due to the historic patient cohort (*n* = 7, 21%). Our findings need to be validated preferably in a prospective clinical trial with routine implementation of NGS testing of all patients.

## Conclusions

The SPI was more accurate than the MCPS to determine patients’ life expectancies and should be recommended for clinical guidance and treatment allocation.The findings need to be validated in a prospective clinical trial with implementation of NGS results in the scoring systems.

## Data Availability

The datasets used and analysed during the current study are available from the corresponding author on reasonable request.
